# B cell activating factor regulates periodontitis development by suppressing inflammatory responses in macrophages

**DOI:** 10.1186/s12903-021-01788-6

**Published:** 2021-09-04

**Authors:** Lixia Wang, Tianyi Zhang, Zheng Zhang, Zihan Wang, Yu-Jie Zhou, Zuomin Wang

**Affiliations:** 1 Department of Stomatology, Beijing Chao-Yang Hospital, Capital Medical University, 8th Gongti South Road, Beijing, 100020 China; 2 International Medical Center, Tianjin Stomatological Hospital, School Medicine, Nankai University, 75th Dagu North Road, Tianjin, 300041 China; 3 Tianjin Key Laboratory of Oral Maxillofacial Function Reconstruction, 75th Dagu North Road, Tianjin, 300041 China; 4 Department of Stomatology, School of Stomatology, Shanxi Medical University, 56 Xinjian South Road, Yingze, Taiyuan, 030001 Shaanxi China; 5 Department of Immunology, School of Basic Medical Sciences, Advanced Innovation Center for Human Brain Protection, Beijing Key Laboratory for Cancer Invasion and Metastasis, Department of Oncology, Capital Medical University, Beijing, China

**Keywords:** Periodontitis, B cell activating factor, Macrophages, *P. gingivalis* LPS

## Abstract

**Background:**

B cell activating factor (BAFF) is a member of the tumor necrosis factor (TNF) superfamily with immunomodulatory effects on both innate and adaptive immune responses. Periodontitis is an inflammatory disease characterized by periodontal soft tissue inflammation and the progressive loss of periodontal ligament and alveolar bone. Macrophages are closely related to periodontitis progression. However, the role of BAFF in periodontitis development and macrophage polarization and the underlying mechanism remain unknown.

**Methods:**

In vivo, a ligation-induced mouse model of periodontitis for BAFF blockade was established to investigate the expression of inducible nitric oxide synthase (iNOS) through real-time PCR (RT-PCR) and immunohistochemistry. In addition, the level of TNF-α in the periodontium, the number of osteoclasts, and alveolar bone resorption were observed. In vitro, RAW 264.7 macrophage cells were treated with 100 ng/mL *Porphyromonas gingivalis* lipopolysaccharide (*P. gingivalis* LPS) in either the presence or absence of 50 nM small interfering RNA (siRNA) targeting BAFF, followed by further incubation for 24 h. These cells and supernatants were collected and stored for RT-PCR, enzyme-linked immunosorbent assay, western blotting and immunofluorescence microscopy.

**Results:**

In vivo, BAFF blockade decreased the levels of TNF-α in the periodontium in a ligature-induced mouse periodontitis model. Reduced osteoclast formation and lower alveolar bone loss were also observed. In addition, BAFF blockade was related to the expression of polarization signature molecules in macrophages. In vitro, BAFF knockdown notably suppressed the production of TNF-α in RAW 264.7 cells stimulated by *P. gingivalis* LPS. Moreover, BAFF knockdown attenuated the polarization of RAW 264.7 cells into classically activated macrophages (M1), with reduced expression of iNOS.

**Conclusions:**

Based on our limited evidence, we showed BAFF blockade exhibits potent anti-inflammatory properties in mice experimental periodontitis in vivo and in *P. gingivalis* LPS-treated RAW 264.7 cells in vitro, and macrophage polarization may be responsible for this effect.

**Supplementary Information:**

The online version contains supplementary material available at 10.1186/s12903-021-01788-6.

## Background

Periodontitis is an inflammatory disease characterized by periodontal soft tissue inflammation and progressive loss of the periodontal ligament and alveolar bone, which may eventually cause tooth loss [1, 2]. Moreover, epidemiological studies have shown that periodontitis is closely associated with an increased risk for several systemic diseases, such as cardiovascular disease, diabetes, pulmonary disease, and preterm birth [3–6]. In the pathogenic progression of periodontitis, plaque microorganisms are the initial factor of periodontitis, and the host immune response determines the process of periodontal tissue destruction [7]. Macrophages are an important part of host immunity as they recognize, phagocytize, and remove foreign pathogens and foreign bodies. They can secrete a variety of cytokines to regulate the immune response and activate adaptive immunity, playing an important role in the immune system. Many studies have shown that macrophages are closely related to the progression of periodontitis [8, 9].

Macrophages are complex cell groups with different subtypes. The functions of different types of macrophages are quite different [10–12]. According to the stimuli and cytokine secretion, macrophages can be divided into classically activated macrophages (M1) and alternatively activated macrophages (M2) [13–15]. M1 macrophages upregulate inducible nitric oxide synthase (iNOS), which generates nitric oxide, CD86, and tumor necrosis factor-α (TNF-α) to maintain the inflammation [16–18]. By contrast, M2 macrophages upregulate arginase 1 (Arg1) expression. They secret high levels of interleukin-10 (IL-10), CD163, and CD206 to promote the resolution of inflammation, wound healing, and other reparative phenomena [14, 16, 19]. Previous studies have shown that both M1 and M2 macrophages exist in periodontal lesions. Periodontal inflammation is related to the accumulation of M1 and M2 macrophages, and the transformation of M2 to M1 macrophages mediates alveolar bone loss [16, 20]. Moreover, *Porphyromonas gingivalis* lipopolysaccharide (*P*. *gingivalis* LPS) stimulation can also promote the polarization of macrophages into the M1 phenotype, which infiltrates into the periodontal tissues and causes alveolar bone resorption [21]. Therefore, the behavior of macrophage may affect the processes of periodontal tissue damage.

B cell activating factor (BAFF), a member of the TNF superfamily, is expressed by macrophages, monocytes, dendritic cells, stimulated neutrophils and stromal cells [22]. BAFF mainly regulates B cell homeostasis and promotes B cell survival and differentiation by binding to its receptor [23, 24]. However, BAFF could also modulate the expression of some inflammatory molecules in RAW 264.7 macrophages and in white adipose tissues of diet-induced obese mice [25]. Using microarray analysis, the top 250 pairs of genes, including the BAFF gene, that may be involved in the interaction of macrophage-associated multiple myeloma cells, were listed [26, 27]. Investigators have reported that BAFF mRNA and protein were upregulated in the early stages of experimental periodontitis in mice [28]. Associations between BAFF and the clinical indices of periodontal disease have been shown in a recent study comparing untreated aggressive and chronic periodontitis, where BAFF correlates with clinical attachment loss (CAL) and clinical probing depth (CPD)[29]. In addition, macrophages are found to be involved in periodontal bone damage through the phenotypic switch of alternatively activated M2 to classically activated M1 [17, 30]. However, the role of BAFF in periodontitis development and macrophage polarization and the underlying mechanism remain unknown.

In this study, we induced experimental periodontitis in mice using ligature and explored the effects of anti-BAFF neutralizing antibody on the periodontal immune response and macrophage polarization in vivo. We investigated the modulatory effects of BAFF knockdown in *P. gingivalis* LPS-treated RAW 264.7 cells to identify the potential mechanism by which BAFF neutralization modulates periodontitis development.

## Methods

### In vivo

#### Animals

Male C57BL/6 mice aged 8–10 weeks were purchased from HuaFukang Bioscience Company (Beijing, China). All the mice used for the experiments were housed in sterile, specific-pathogen-free units with free access to food and water during the entire experimental period. The room temperature was maintained at 21–23 °C, and the relative humidity in the room was 45–55%. The mice were maintained on a 12-h dark–12-h light cycle. The animal protocols used in this study were approved by the Capital Medical University Animal Care and Use Committee (No. AEEI-2020-067).

#### Experimental protocols

First, to explore the local expression of BAFF in experimental periodontitis, mice were divided into two experimental groups: a control group (n = 5) and a periodontitis group (n = 5). To induce experimental periodontitis in mice, a ligature-induced experimental periodontitis model was used as previously described, with a slight modification [31]. Briefly, a 5-0 silk suture (Mersilk, Ethicon, UK), approximately 1 cm long, was placed around the maxillary second molar in the gingival sulcus and tied using a surgeon’s knot at the distal-palatal line angle. In addition, the ligatures were also placed around the teeth in control group to eliminate the influence of the probable tissue destruction caused by mechanical trauma, but they were quickly removed once they are put in place. Two weeks after ligature placement, the mice were sacrificed and the tissues were harvested to determine BAFF expression.

**Fig. 1:**
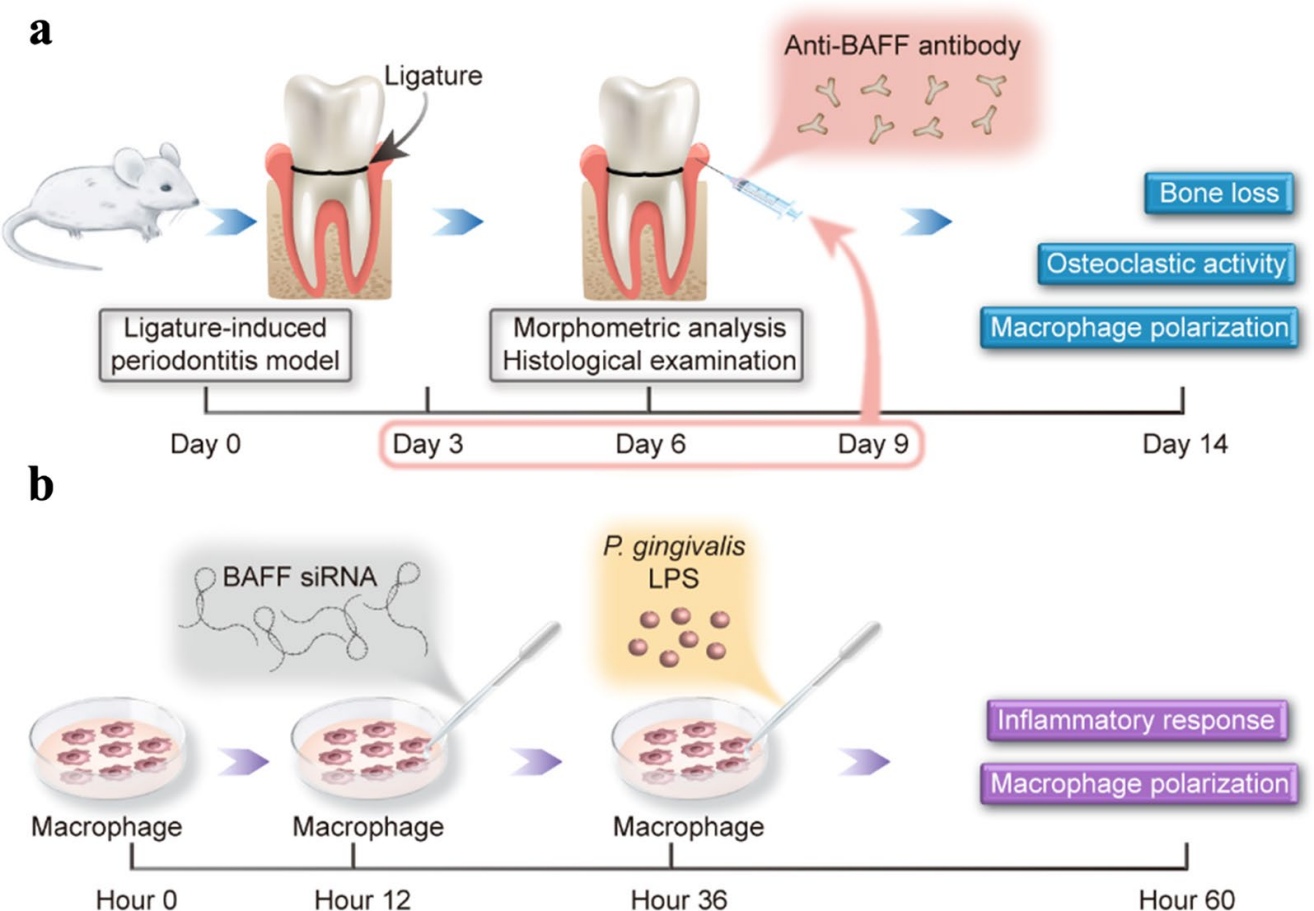
Schematic diagram showing the design of the experiment. **a** Schematic diagram of BAFF blockade method in the mouse periodontitis model. **b** Schematic diagram of RAW 264.7 cells processing method

Based on the results of the aforementioned procedure, we conducted a short-term experiment to estimate the effect of an anti-BAFF neutralizing antibody on macrophage infiltration and periodontal tissue destruction in mice (Fig. 1a). The mice were randomly divided into three experimental groups: control (Isotype) group (n = 5), periodontitis (Isotype) group (n = 5), and periodontitis (Anti-BAFF) group (n = 5). Anti-BAFF neutralizing antibody was used for BAFF blockade in a periodontitis mouse model. Briefly, after the induction of periodontitis, mice were injected with either 5 μl of anti-BAFF antibody (Sandy-2; IgG1; Adipogen, 1 mg/ml) or an equal amount of isotype control IgG (R&D, MAB005) into the palatal gingival papilla between the first and second molars and the second and third molars of each mouse on day 3, 6, and 9 [28, 32]. For all procedures, the mice were anesthetized with 4% chloral hydrate (0.2 mL/20 g; Tianjin Guangfu Fine Chemical Research Institute, Tianjin, China) via peritoneal injection. After 14 days, all mice were sacrificed and the maxillaries were harvested to estimate the effect of the anti-BAFF neutralizing antibody on macrophage polarization and periodontal tissue destruction.

#### Bone morphometric analysis

The maxillae of mice were defleshed, bleached with 3% hydrogen peroxide and stained with 1% toluidine blue. Bone resorption was assessed at 40× using digital stereomicroscopy. Images of the palatal surfaces of the maxillae were captured, and the polygonal area of bone loss was measured using ImageJ software (NIH). The area was enclosed longitudinally from the cementoenamel junction (CEJ) to the alveolar bone crest (ABC) and transversely from the area distal to the first maxillary molar to the area mesial of the third maxillary molar, as previously described [32, 33]. The results are presented in square millimeters (n = 5 animals/group). All alveolar bone resorption measurements were performed by two blinded evaluators.

#### Histology and immunohistochemical analysis

The collected maxillae were fixed in 4% formaldehyde overnight, followed by decalcification in 10% EDTA for four weeks at 4 °C, and dehydrated in graded ethanol. Paraffin-embedded specimens were cut into of 5 μm-thick sections along the long axis of the molars. Moreover, the tissue sections in the mesio-distal direction were prepared according to a reported method [32]. After dewaxing and rehydration, the sections were stained with hematoxylin and eosin (HE) or subjected to immunohistochemical analysis with rabbit polyclonal anti-iNOS (1:200, Cat number: ab15323, Abcam, Cambridge, UK) and rabbit polyclonal anti-CD45 (1:1500, Cat number: GB11066, Solarbio, Beijing, China). The quantitative analysis of iNOS and CD45 expression in the periodontal tissues around maxillary second molars was accomplished using Image Pro Plus 6.0 software. Inflammatory cell infiltration of the periodontal tissues was determined as described previously [34–36]. Briefly, the total number of inflammatory cells, such as monocyte-macrophage system and lymphocytes, was manually counted according to their morphology, from 3 separate fields on HE staining sections (400× magnification). The number of CD45-positive cells was calculated according to the method of Wu et al. [36]. The four square fields (100 × 100 μm^2^) of connective tissue adjacent to the junctional epithelium indicate target areas were used to count CD45-positive cells (n = 5), and three counted outcomes were averaged for each section. Data were expressed as the mean number of cells per 100 × 100 µm^2^ of connective tissue.

#### Tartrate-resistant acid phosphatase staining (TRAP)

The maxillary sections of mice were subjected to TRAP staining using a commercial kit (Solarbio, Beijing, China) according to the manufacturer’s instructions. The number of osteoclasts per 500 × 500 squaremicrometers around the alveolar bone surface was counted and analyzed using TRAP staining images.

#### Quantitative real-time PCR(RT-PCR)

Total RNA was extracted from mouse gingival tissue samples using the FastPure Cell/Tissue Total RNA Isolation Kit (Vazyme Biotech Co., Ltd). cDNA was generated using a HiScript® III RT SuperMix for qPCR (+ gDNA wiper) (Vazyme Biotech Co., Ltd) according to the manufacturer’s protocol. Quantitative RT-PCR was performed using 10 μL 2 × AceQ® Universal SYBR Green qPCR Master Mix (Vazyme Biotech Co., Ltd), 0.4 μl forward primer, 0.4 μL reverse primer, and 2 μL cDNA in a 20 μL total volume. All samples were subjected to denaturation for 5 min at 95 °C, followed by 40 cycles of 95 °C for 10 s and 60 °C for 30 s. Expression data were normalized to the amount of glyceraldehyde­3-phosphate dehydrogenase (GAPDH) mRNA using the − ΔΔCt method. The primer sequences for each gene are listed in Table 1.

**Table 1:** Primers used for real-time PCR

Genes	Forward primers 5′-3′	Reverse primers 5′-3′
GAPDH	AGAAGGTGGTGAAGCAGGCATC	CGAAGGTGGAAGAGTGGGAGTTG
BAFF	CCACCGTGCCTCTGTTTTTG	CTTCTGCGGAGTGATGGGAT
RANKL	AGGCTGGGCCAAGATCTCTA	GTCTGTAGGTACGCTTCCCG
OPG	ACCCAGAAACTGGTCATCAGC	CTGCAATACACACACTCATCACT
iNOS	GTTCTCAGCCCAACAATACAAGA	GTGGACGGGTCGATGTCAC
Arg1	CTCCAAGCCAAAGTCCTTAGAG	GGAGCTGTCATTAGGGACATCA
TNF-α	AAAGGGGATTATGGCTCAGG	CTCCCTTTGCAGAACTCAGG
IL-10	GCTCTTACTGACTGGCATGAG	CGCAGCTCTAGGAGCATGTG

### In vitro study

#### Cell culture

RAW 264.7 cells, obtained from the China National Infrastructure of Cell Line Resource (Beijing, China), were routinely cultured in Dulbecco’s modified Eagle’s medium (DMEM) (Gibco, Thermo Fisher Scientific, Waltham, USA) containing 10% fetal bovine serum, 100 units/mL penicillin, and 100 units/mL streptomycin at 37 °C in a 5% CO_2_ atmosphere as previously reported [37].

#### RNA interference and transfection

The siRNAs targeting BAFF and siRNA NC were all purchased from RiboBio. The siRNA sequences were si­BAFF­1: CTGAAACACTACCCAATAA, si-BAFF-2: TCACGGTGGTGTCTTTCTA and si-BAFF-3: CCAACTTGCAATACCAAGA. Transfection was performed using 5 μL (per six­well plate) of either si­BAFF or siRNA NC and 12 μL ribo*FECT*™ CP Reagent (RiboBio, Guangzhou, China) at a final concentration of 50 nM. Twenty-four hours post­transfection, the cells were collected to evaluate their interference efficiency. We selected si­BAFF­3 for the further study.

#### Effects of silencing BAFF on macrophage polarization and inflammatory response

To investigate the effects of BAFF on the inflammatory response and phenotype switch of macrophages, experimental wells were treated with *P*. *gingivalis* LPS (100 ng/mL) (Invivogen, San Diego, CA, USA) in either the presence or absence of si-BAFF (50 nM) followed by further incubation for 24 h. These cells and supernatants were collected and stored for RT-PCR, enzyme-linked immunosorbent assay (ELISA), western blot assays and immunofluorescence microscopy (Fig. 1b).

#### RT-PCR

Total RNA isolated from cells was extracted using FastPure Cell/Tissue Total RNA Isolation Kit (Vazyme Biotech Co., Ltd) according to the manufacturer’s instructions and reverse transcribed into cDNA. The gene expression of factors related to the M1 phenotype (iNOS and TNF-α) and the M2 phenotype (Arg1 and IL-10) were analyzed using 2 × AceQ® Universal SYBR Green qPCR Master Mix (Vazyme Biotech Co., Ltd). The primer sequences for each gene are listed in Table 1.

#### Enzyme­linked immunosorbent assay (ELISA)

The levels of TNF-α and IL-10 in the cell supernatants were detected using an ELISA kit (Nanjing Jiancheng Bioengineering Institute, China) in accordance with the manufacturer’s instructions.

#### Western blotting

Cells treated for 24 h were collected and used for western blotting to assess the expression of iNOS (M1 marker) and Arg1 (M2 marker) protein. Cells were harvested and washed twice with ice-cold phosphate-buffered saline (PBS). RIPA lysis buffer (Beyotime Biotechnology) was used to extract the total proteins. Protein concentrations were measured by using an Enhanced BCA Protein Assay Kit (Beyotime Biotechnology, Haimen, China) according to the manufacturer’s instructions. The proteins were separated by SDS-PAGE and subsequently transferred onto polyvinylidene fluoride membranes (Millipore, Bedford, MA, USA). Membranes were blotted with antibodies against iNOS (diluted 1:250, rabbit, Abcam), Arg1 (diluted 1:1000, goat, Abcam), β-actin (diluted 1:1000, rabbit, Beyotime) and horseradish peroxidase (HRP) -conjugated secondary antibodies (diluted 1:1000, CWBiotech, Beijing, China). The protein bands were visualized using BeyoECL Plus (Beyotime) according to the manufacturer’s instructions and exposed to X-ray film. The ratio of the intensity of the target protein to that of β-actin loading control was calculated to represent the expression level of the protein.

#### Immunofluorescence microscopy

The RAW 264.7 cells were grown on glass coverslips, and immunofluorescence assay was performed [38]. The cells were incubated with antibodies specific for either iNOS (diluted 1:250, Rabbit, Abcam) or CD206 (diluted 1:1000, Rabbit, Abcam) followed by the appropriate secondary antibodies Cy3 (diluted 1:1000, goat, Beyotime), and then further incubated with DAPI for 5 min. The cells were visualized by using laser scanning confocal microscope (Leica TCSSP5).

### Statistical analysis

All statistical computations were performed using GraphPad Prism software (version 8.0), and the statistical significance was analyzed using unpaired two-tailed Student’s t test or one-way analysis of variance (ANOVA) with Tukey’s post hoc multiple-comparison test unless stated otherwise. All data are presented as mean ± standard deviation (SD). Pearson product-moment correlation was used for the correlation analysis. Differences were considered significant at *P* < 0.05.

## Results

In this study, we established an experimental periodontitis mouse model to assess the effect of local injection of anti-BAFF antibody into the gingiva on the process of periodontal inflammation. To better understand the immunomodulatory role of BAFF, RAW 264.7 cells were stimulated with *P. gingivalis* LPS in either the presence or absence of BAFF. The polarization signature molecules and cytokines expressed by the cells were then detected in an in vivo study.

### Expression of BAFF in a periodontitis mouse model

**Fig. 2:**
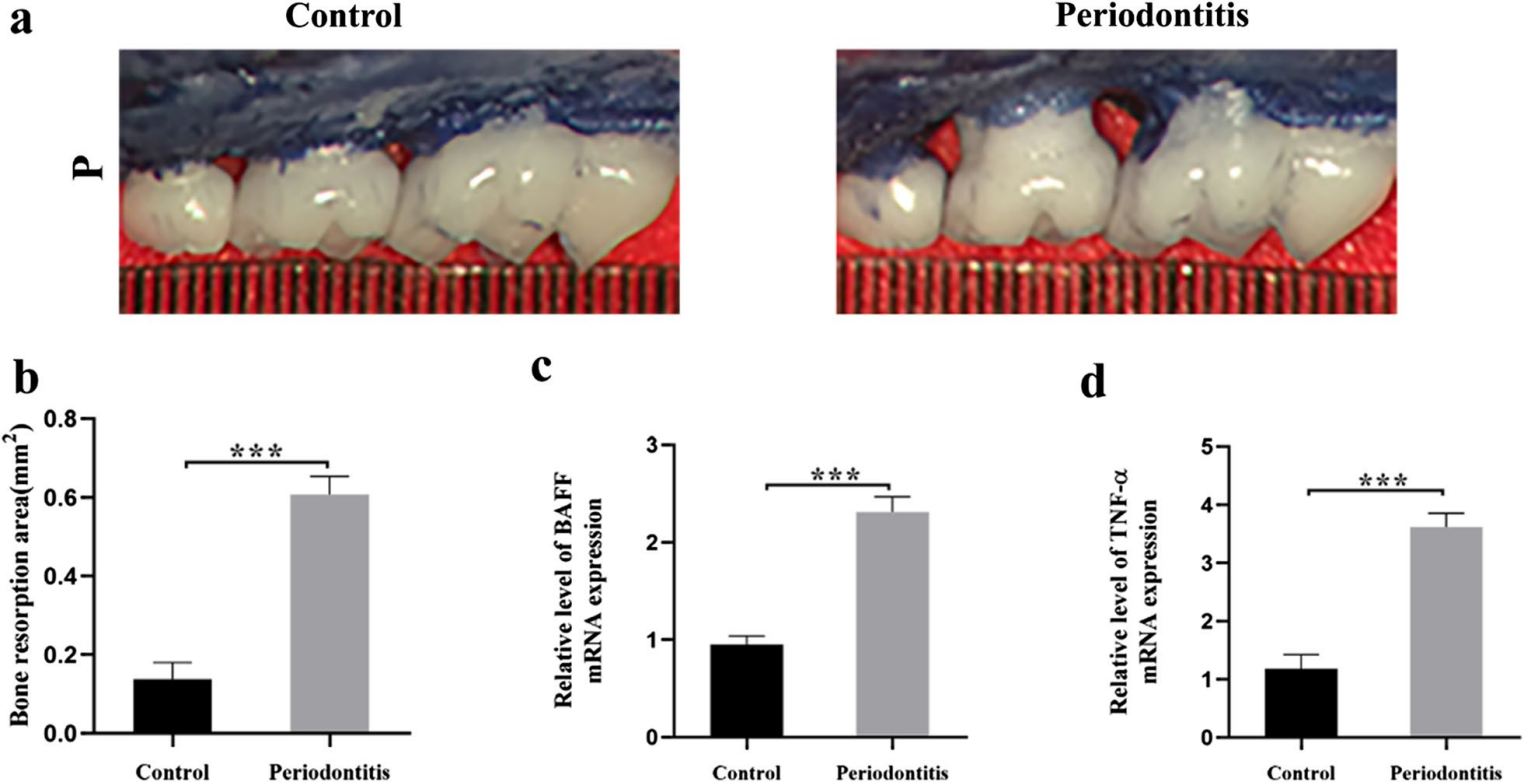
Expression of BAFF in experimental periodontitis in mice. Ligature was placed around the maxillary right second molar and left second molar was used as a control side. **a** Representative images from experimental periodontitis as indicated by alveolar bone loss. P, palatal surface. **b** Quantification analysis of alveolar bone resorption. **c**, **d** Expression of BAFF and TNF-α was measured by real-time PCR. Data are presented as mean ± standard deviation. ***P < 0.001 using two-tailed Student’s t-test

To evaluate the involvement of BAFF in the periodontitis model, we first developed a periodontitis mouse model as described in the Methods section (Fig. 2a), and the expression of BAFF was determined on day 14 after the induction. The bone area at the palatal groove site of the second maxillary molar (Fig. 2b) was measured. Significant alveolar bone loss was observed in the mice in the periodontitis group. The levels of BAFF mRNA expression were significantly higher in gingival tissues from mice with periodontitis than in controls (Fig. 2c). We also found that the TNF-α mRNA expression was significantly increased in the periodontitis group compared to that in the control group. (Fig. 2d).

### Effect of BAFF inhibition on alveolar bone resorption and the number of osteoclasts in periodontium

**Fig. 3:**
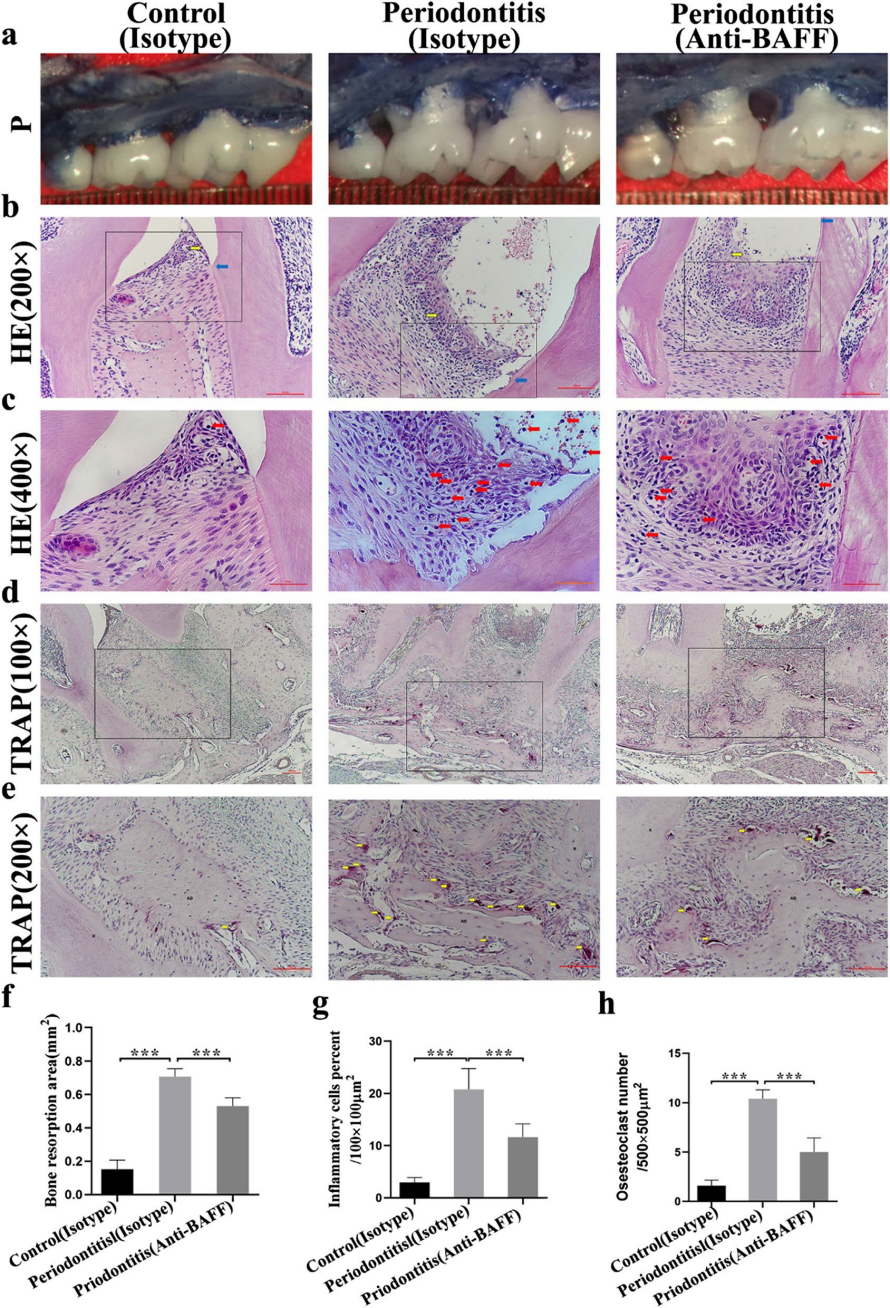
Effect of BAFF inhibition on alveolar bone loss and osteoclast formation. **a** Representative images of alveolar bone resorption from ligature mice model. P, palatal surface. **b**, **c** Representative images of HE staining of the periodontal tissue from ligature mice model. Blue arrows, cemento-enamel junction (CEJ); yellow arrows, junctional epithelium (JE). Final magnification 200×, 400×. **d**, **e** Representative images of TRAP staining from ligature mice model. Arrows indicate TRAP-positive osteoclasts. R, root; AB, alveolar bone. Final magnification 100×, 200×. **f** Bone resorption area was calculated from cemento-enamel junction to the alveolar bone crest of the second maxillary molar at palatal groove site. **g** The percent of inflammatory cell infiltration in the periodontal tissues of mice was countered after HE staining. **h** Comparison of osteoclasts number/500 × 500 µm^2^ of bone surface area among groups. Data are presented as mean ± standard deviation. ***P < 0.001 using one-way ANOVA with Tukey's test. *ANOVA* analysis of variance, *TRAP* tartrate-resistant acidic phosphatase

**Fig. 4:**
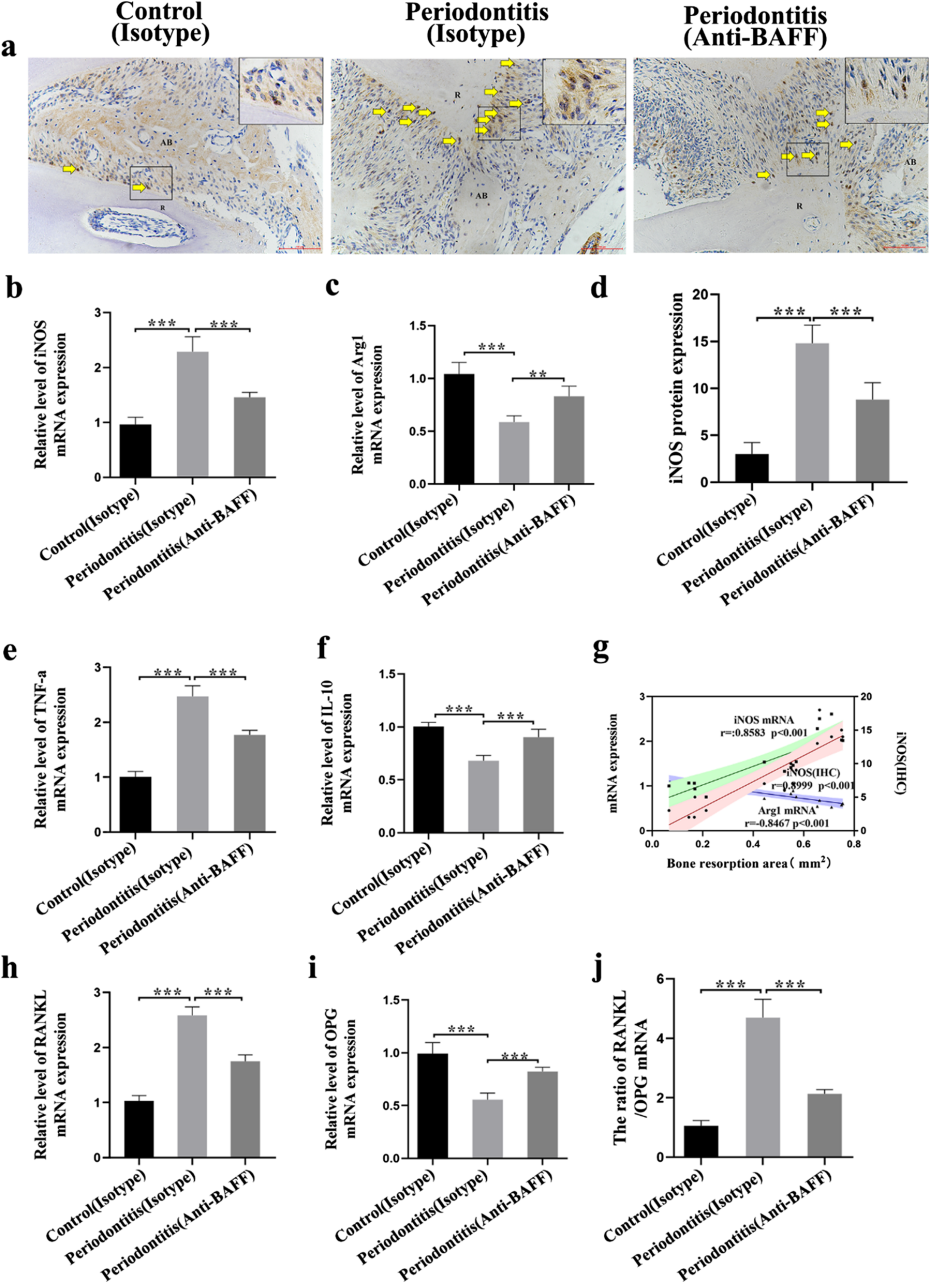
Expression of polarization signature molecules in periodontal tissues of mice. **a** Representative immunohistochemistry micrographs of periodontal tissues of maxillary second molars identifying the M1 macrophage marker iNOS. Arrows indicate areas of high expression of iNOS. R, root; AB, alveolar bone. Magnification: 200×. Scale bar, 100 μm. **d** The corresponding quantitative analysis of iNOS expression in periodontal tissues. **b**, **c**, **e**, **f** Real-time PCR analysis of the mRNA expression of the M1-related iNOS and TNF-α and the M2-related Arg1 and IL-10 in gingival tissues. **g** Correlations between bone resorption area and mRNA expression of iNOS and Arg1 and protein of iNOS (Pearson). **h**, **i**, **j** Expression of receptor activator of nuclear factor κB ligand (RANKL), osteoprotegerin (OPG) mRNA, and the ratio of RANKL/OPG in gingival tissues, as determined by RT-PCR. Data are presented as mean ± standard deviation. **P < 0.01 and ***P < 0.001 using one-way ANOVA with Tukey’s test

To assess the effect of BAFF inhibition on pathological bone resorption and the number of osteoclasts in the periodontium in vivo, we administrated a local injection of anti-BAFF antibody or isotype control antibody in the gingiva in a ligature-induced experimental periodontitis model (Fig. 3a, f). As shown in Fig. 3f, the anti-BAFF antibody-treated group with periodontitis (anti-BAFF-periodontitis group) showed significantly reduced alveolar bone resorption when compared with the periodontitis group treated with isotype control antibody (isotype-periodontitis group). In the ligature-induced periodontitis model, we observed that the number of osteoclasts was decreased significantly in the anti-BAFF-periodontitis group when compared with the isotype-periodontitis group (Fig. 3d, e, h). In addition, the expression of the factors related to osteoclastogenesis activity, including receptor activator of nuclear factor κB ligand (RANKL) and osteoprotegerin (OPG), in the periodontium were also determined (Fig. 4h, i). We found that the expression of RANKL mRNA was increased in the periodontitis group (isotype-periodontitis group), while the expression of OPG was decreased. Moreover, anti-BAFF antibody played an inhibitory role against the RANKL/OPG ratio change in the inflammatory microenvironment, with significantly reduced the mRNA levels of RANKL and elevated the expression of OPG (Fig. 4j).

To assess inflammatory infiltration, HE staining and immunohistochemical staining for CD45 (also known as leukocyte common antigen) were performed. Very few inflammatory cells were present around the connective tissue in the control group treated with isotype control antibody (isotype-control group) (Fig. 3b, c, g; Additional file 1: Supplementary Fig. 2a and b). Compared with the Control group, all periodontitis groups with ligature placement had significantly increased numbers of CD45-positive cells. The application of anti-BAFF antibody significantly downregulated this inflammatory response and the level of CD45 in the periodontal tissues (Fig. 3b, c, g; Additional file 1: Supplementary Fig. 2a and b).

### Effect of BAFF inhibition on the expression of periodontitis-related and macrophage-related molecules

According to the results of our immunohistochemical analysis and RT-PCR, the expression of iNOS in the isotype-periodontitis group was significantly higher than that in the control group treated with isotype control antibody (isotype-control group) (Fig. 4a, b, d). The application of anti-BAFF antibody significantly downregulated this inflammatory response and the level of iNOS in the periodontal tissue (Fig. 4b, d). In addition, we found that the mRNA expression of TNF-α was significantly increased in the isotype-periodontitis group compared to that in the control group (Fig. 4e). TNF-α expression level was notably reduced after treatment with the anti-BAFF antibody. Furthermore, anti-BAFF antibody treatment also significantly increased local IL-10 mRNA levels (Fig. 4f). Finally, there was a moderate but significant positive correlation between alveolar bone resorption severity and the level of iNOS (r = 0.8999 [IHC], r = 0.8583 [RT-PCR], *P* < 0.001, n = 15; Fig. 4g). A moderate increase in Arg1 mRNA in the anti-BAFF-periodontitis group was also observed in the ligature-induced model (Fig. 4c), and the negative correlation between alveolar bone resorption severity and the level of Arg1 mRNA was to a statistically significant extent (r =  − 0.8467, *P* < 0.001, n = 15; Fig. 4g).

### BAFF knockdown exhibited anti-inflammatory effects in *P*.* gingivalis* LPS-stimulated macrophages

**Fig. 5:**
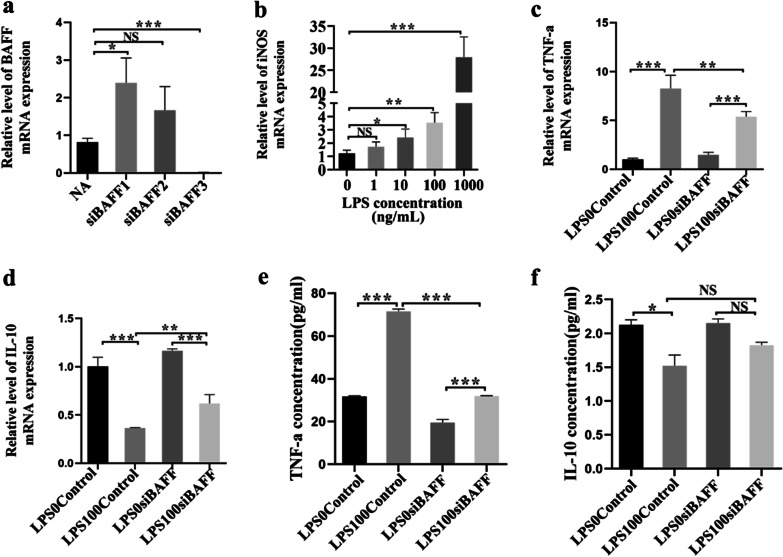
BAFF knockdown inhibited the LPS-induced inflammatory response of macrophages and promoted the macrophage phenotype switch from M1 to M2. RAW 264.7 cells were treated with or without BAFF siRNA for 24 h. **a** siRNA knockdown of BAFF is confirmed by real-time PCR. **b** Real-time PCR was used to measure the level of iNOS by applying different concentrations of *P. gingivalis* LPS (0 ng/mL, 1 ng/mL, 10 ng/mL, 100 ng/mL, 1000 ng /mL) in RAW 264.7 cells for 24 h. The level of iNOS was upregulated by applying of 100 ng/mL *P*. *gingivalis* LPS in RAW 264.7 cells. **c**, **d** Real-time PCR analysis of the gene expression of the M1-related TNF-α and the M2-related IL-10. **e**, **f** Concentrations of TNF-α and IL-10 in the cell supernatant measured by ELISA. Data are shown as the mean ± SD of three independent experiments. NS, the difference was not significant,*P < 0.05, **P < 0.01 and ***P < 0.001 using one-way ANOVA with Tukey's test

Our studies have suggested that the inhibition of anti-BAFF antibody could decrease alveolar bone resorption and M1 phenotype polarization. Therefore, to further explore the importance of BAFF in macrophage polarization, we designed three siRNAs, and the interference efficiency of BAFF was detected by RT-PCR (Fig. 5a). Based on these data, we selected si­BAFF-3 together with 100 ng/mL *P. gingivalis* LPS to investigate the immunoregulatory effects of BAFF on the inflammatory response and polarization of macrophages (Fig. 5a, b). During inflammation resulting from *P. gingivalis* LPS, BAFF knockdown showed great anti-inflammatory property. According to the RT-PCR results, the LPS stimulation led to extremely high expression of TNF-α and low levels of IL-10, and this effect was remarkably inhibited by BAFF knockdown (Fig. 5c, d). The ELISA results for TNF-α were consistent with those of RT-PCR (Fig. 5e). The level of IL-10 protein tended to increase in the LPS100-siBAFF group compared to the LPS100-control group; however, the differences were not significant (Fig. 5f).

### BAFF knockdown modulated macrophage polarization upon *P. gingivalis* LPS stimulation

**Fig. 6:**
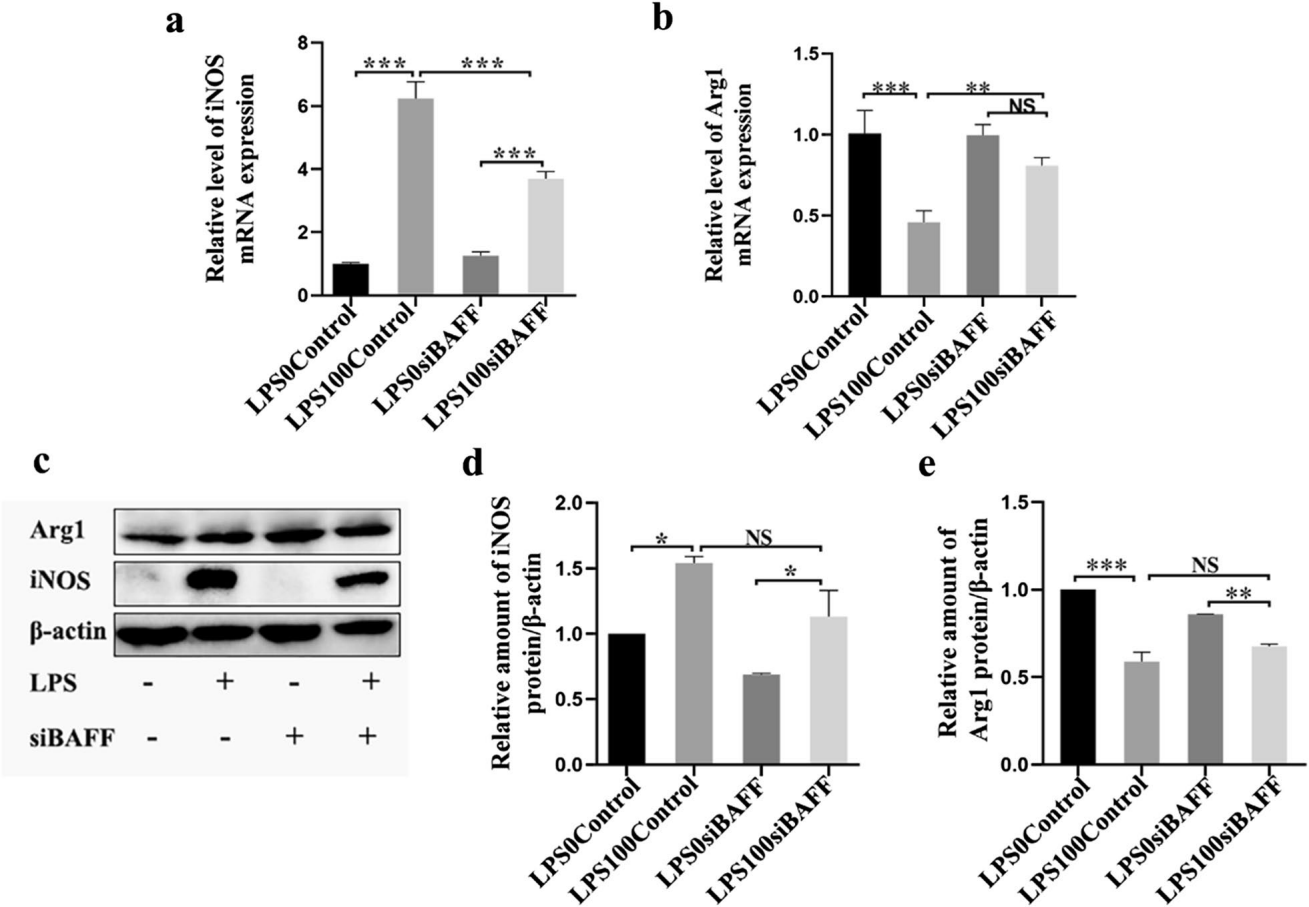
BAFF knockdown altered polarization signature molecule expression in macrophages. **a**, **b** Real-time PCR analysis of the mRNA expression of the M1-related iNOS and the M2-related Arg1. **c**, **d**, **e** Relative protein levels of iNOS and Arg1determined by western blotting. Data are presented as mean ± standard deviation. Final magnification 1000×. NS, the difference was not significant, *P < .05, **P < .01 and ***P < .001 using one-way ANOVA with Tukey's test

**Fig. 7:**
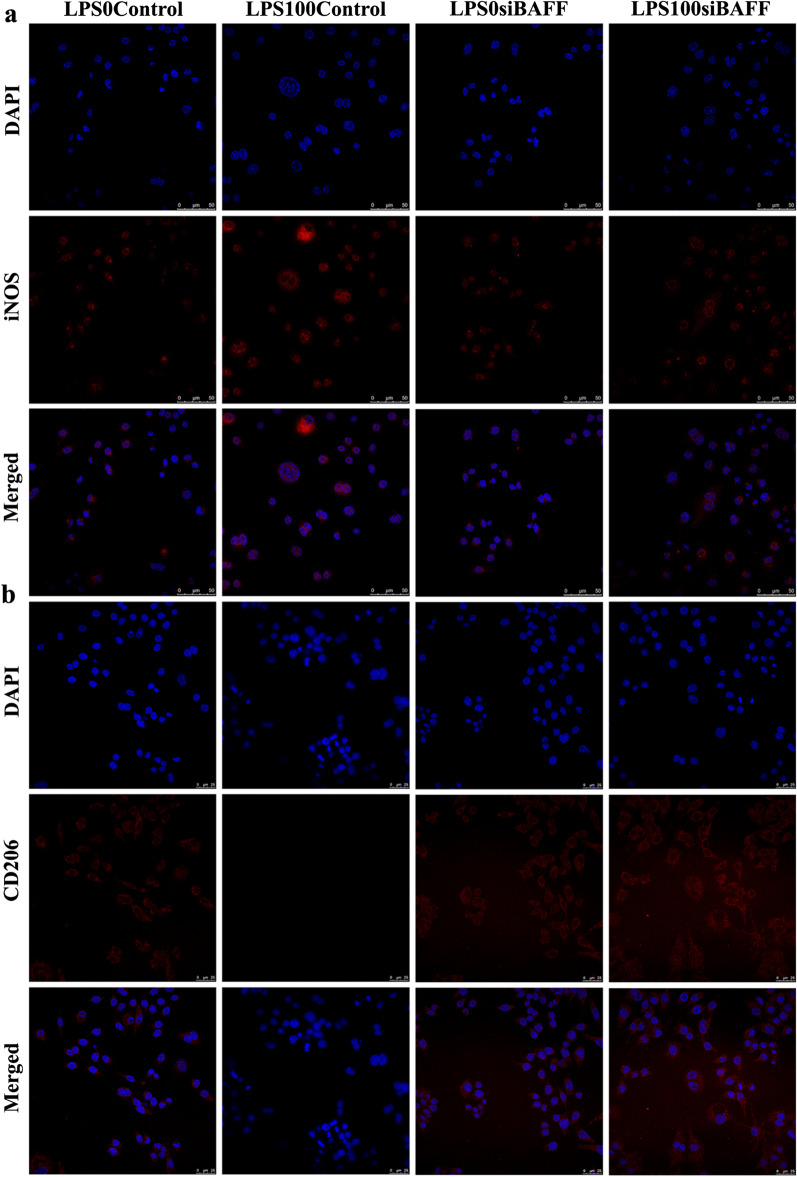
BAFF knockdown promoted the macrophage phenotype switch from M1 to M2. **a** Representative immunofluorescence staining of M1 phenotype macrophages (iNOS+; red), BAFF knockdown reduced the expression of iNOS which was elevated by *P*. *gingivalis* LPS. **b** Representative immunofluorescence staining of M2 phenotype macrophages (CD206+; red). BAFF knockdown facilitated the expression of CD206 which was decreased by *P*. *gingivalis* LPS. The nuclei were stained with DAPI (blue). *P*. *gingivalis* LPS, *Porphyromonas gingivalis* lipopolysaccharide

Macrophage phenotype was determined by RT-PCR, western blotting and immunofluorescence. *P. gingivalis* LPS significantly upregulated the levels of iNOS mRNA compared to the control group (LPS0-control group) (Fig. 6a). In the presence of LPS stimulation, BAFF knockdown not only decreased the mRNA levels of M1-related factors such as iNOS, but also lowered the expression of M2-related factors, including Arg1 in macrophages (Fig. 6a, b). Moreover, western blotting (Fig. 6c, d, e) showed that *P. gingivalis* LPS aggravated the generation of the M1 marker iNOS and attenuated the formation of the M2 marker Arg1, and BAFF knockdown inhibited the phenotypic switch in this process. Furthermore, as shown in Fig. 7a, b, BAFF knockdown, induced by *P*. *gingivalis* LPS, decreased the M1 phenotype ratio (iNOS+, red) and increased the M2 phenotye ratio (CD206+, red). Taken together, these results indicate that BAFF plays an important role in the induction of M1 phenotype in RAW 264.7 cells.

## Discussion

BAFF is a new member of the tumor necrosis factor superfamily discovered by Moore [22], and it widely exists in gingival tissue, gingival crevicular fluid, saliva and serum, and its expression changes in subjects with periodontal diseases [28, 39, 40]. BAFF could strongly induces monocyte survival, activation, and differentiation into macrophage-like cells, as measured by proinflammatory cytokine secretion and upregulation of costimulatory molecule expression [41]. Previous studies have suggested that macrophages participate in the immune response, which is essential in the initial host defense system during periodontal pathological progress, and are critical for inflammation and tissue repair [20, 30]. According to Kim et al., BAFF can regulate macrophages and increase nitric oxide synthase and IL-6 expression in adipose tissue [25]. However, the role of BAFF in periodontitis development and macrophage polarization and the underlying mechanism remain unknown.

Recently, the presence of BAFF in serum and saliva of patients with chronic periodontitis and healthy controls was reported in a cross-sectional study [40]. However, the levels of cytokines in the gingiva can be produced locally, which is not necessarily related to their concentrations in serum or saliva. Our data indicated that BAFF levels are elevated in mice with periodontal bone loss, which is consistent with earlier studies using a similar mouse periodontitis model [28]. Although the authors linked the absence of B cells to the protection of bone loss, they did not monitor the presence of macrophages in the mice [28]. In conclusion, our observations and interventional studies suggest that BAFF blockade and macrophage polarization are temporally and causally related to periodontal bone loss.

In this study, to assess the effect of BAFF inhibition on inflammatory response and pathological bone resorption in the periodontium in vivo, we administrated a local injection of anti-BAFF antibody in the gingiva in a ligature-induced experimental periodontitis model. Periodontal pocket delivery is widely used in clinical settings. Antibiotics and other molecules spread into inflamed tissues through the epithelium and take effect. Our data demonstrated that BAFF blockade suppressed periodontal inflammation and alveolar bone loss in a ligature-induced experimental periodontitis model. Interestingly, TNF-a is an important factor of M1 macrophages, and its activation is known to stimulates the degradation of the connective tissue matrix, activation of osteoclasts, and resorption of bone [42–44]. According to Gaddis et al. [45], IL-10 is important for the control of infection and the progression of periodontitis. When biofilm invasion and host response are unbalanced, excessive inflammatory cytokines such as TNF-α are released to destroy periodontal tissues [46–48]. In this study, it was observed that in situ the expression of TNF-α was significantly decreased and the expression of IL-10 was significantly increased in BAFF blockade mice with periodontal disease. These results suggest that BAFF blockade has an anti-inflammatory effect on periodontitis. In addition, to determine the underlying potential mechanism in vitro, we also examined the anti-inflammatory effect of BAFF knockdown on *P*. *gingivalis* LPS-stimulated macrophages. The expression levels of TNF-α stimulated by *P*. *gingivalis* LPS were suppressed by BAFF knockdown, which was consistent with the results of BAFF blockade in mouse periodontitis model.

The ligature-induced mouse periodontitis model is known for its quick and aggressive alveolar bone loss after ligature placement [31], and it allowed us to observe the emergence of macrophages in the late stages of the experimental bone-loss period. However, animal models have limitations that do not allow the investigators to faithfully reproduce the complexity of a naturally chronic periodontitis lesion. After ligature placement around the cervical region of the second molar, a large amount of plaque and sulcular epithelium ulceration is induced [49]. This action causes the host immune response that leads to inflammatory cell infiltration into the gingival tissue and bone resorption [50]. These results indicate that experimental periodontitis was successfully established in this study.

Considering that it is more reliable to measure bone loss on the palatal side [31], we evaluated the bone loss at the palatal sulcus in each group in the present study. Less alveolar bone loss was observed after anti-BAFF antibody into the periodontal tissues. In order to understand the mechanisms of osteoclast differentiation and formation, RT-PCR was used to detect osteoclastogenesis-related genes in gingival tissues. In periodontitis group, RANKL was upregulated and OPG was downregulated compared with healthy periodontal tissues, leading to the increase of RANKL/OPG ratio, which is the key factor of bone resorption [51, 52]. In this study, BAFF blockade had attenuation of the RANKL/OPG ratio compared with the periodontitis (isotype) group. Therefore, inhibition of RANKL expression by BAFF blockade may be an important mechanism underlying the reduction in differentiation and formation of osteoclast, and bone loss in experimental periodontitis.

In a previous study, CD68+ cells were discovered in inflammatory periodontal tissues [9], suggesting that macrophages play an important role in the innate immune response. However, they may also lead to tissue destruction in periodontal disease [53, 54]. Indeed, we confirmed that local administration of anti-BAFF antibody decreased the number of M1 phenotype macrophages in the ligature-induced periodontitis models. In addition, it has also been reported that *P*. *gingivalis* LPS can polarize macrophages into proinflammatory M1 phenotype and characterize them as proinflammatory [16]. In this study, M1 macrophage polarization induced by *P*. *gingivalis* LPS was inhibited by BAFF knockdown. In addition, BAFF knockdown could induce the differentiation of M2 macrophage. M2 macrophages secreted IL-10 at a high level to promote wound healing [55, 56]. In the context of cell phenotype, macrophage polarization might be part of the mechanisms of tissue inflammation induction and resolution [11, 57]. The mechanism by which macrophages promote wound healing involves two aspects: inhibiting inflammation, and promoting matrix deposition through L-arginine metabolism [58, 59]. Therefore, the multiple regulatory effects of BAFF blockade on the polarization homeostasis of macrophages is conducive to its anti-inflammatory ability.

This study was limited by using only a single time point for inducing periodontitis because macrophages might differ in their functions at different stages of periodontitis [17]. Therefore, an exploration of the dynamics of macrophage phenotypes in periodontitis is required in future studies. Additionally, the effect of BAFF blockade on M1/M2 paradigm is primarily based on animal and in vitro studies, and the precise manifestation of macrophage phenotypes in humans is expected to differ substantially from that in animals [60, 61]; therefore, more investigations of the human version of periodontitis are required.

## Conclusions

Although it is well known that macrophages are critical in periodontitis pathogenesis, and BAFF is a major mediator of their function, to the best of our knowledge, no previous interventional study has investigated the role of BAFF in periodontitis development through macrophages. The present study provides insight into the effects of BAFF blockade on the progression of periodontitis aggravated by ligature, which may provide a novel therapeutic strategy for clinical application. Downregulation of proinflammatory cytokines in macrophages and inhibition of the M1 phenotype are pivotal for the protective effect of BAFF blockade. Notably, BAFF expression levels vary in different periodontal diseases. Thus, in addition to the short-term effect of BAFF blockade on periodontitis, long-term investigations are required to confirm the ability of BAFF blockade to protect the periodontium through macrophages.

## Supplementary Information

**Additional file 1.** The original, unprocessed images of Western blotting and expression of CD45-positive cells in periodontal tissues of mice.

## Data Availability

The data and materials used in the present study are available from the corresponding authors on reasonable request.

## Abbreviations

ABC: Alveolar bone crest
ANOVA: One-way analysis of variance
Arg1: Arginase 1
BAFF: B cell activating factor
CEJ: Cementoenamel junction
DMEM: Dulbecco’s modified Eagle’s medium
ELISA: Enzyme­linked immunosorbent assay
HE: Hematoxylin–eosin staining
iNOS: Inducible nitric oxide synthase
OPG: Osteoprotegerin
PBS: Phosphate buffer saline
*P*. *gingivalis* LPS: *Porphyromonas gingivalis* Lipopolysaccharide
RT-PCR: Real-time polymerase chain reaction
RANKL: Nuclear factor κB ligand
SD: Standard deviation
TNF-α: Tumor necrosis factor-α
TRAP: Tartrate-resistant acid phosphatase
