# Supplementary material for: B cell activating factor regulates periodontitis development by suppressing inflammatory responses in macrophages
Source: BMC Oral Health. 2021 Sep 4;21:426. doi: 10.1186/s12903-021-01788-6 (PMC8418735; doi:10.1186/s12903-021-01788-6)
Supplement: Supplementary file 1 — Additional file 1. The original, unprocessed images of Western blotting and expression of CD45-positive cells in periodontal tissues of mice. [file 12903_2021_1788_MOESM1_ESM.docx]

**Supplementary Appendix.**

**a**


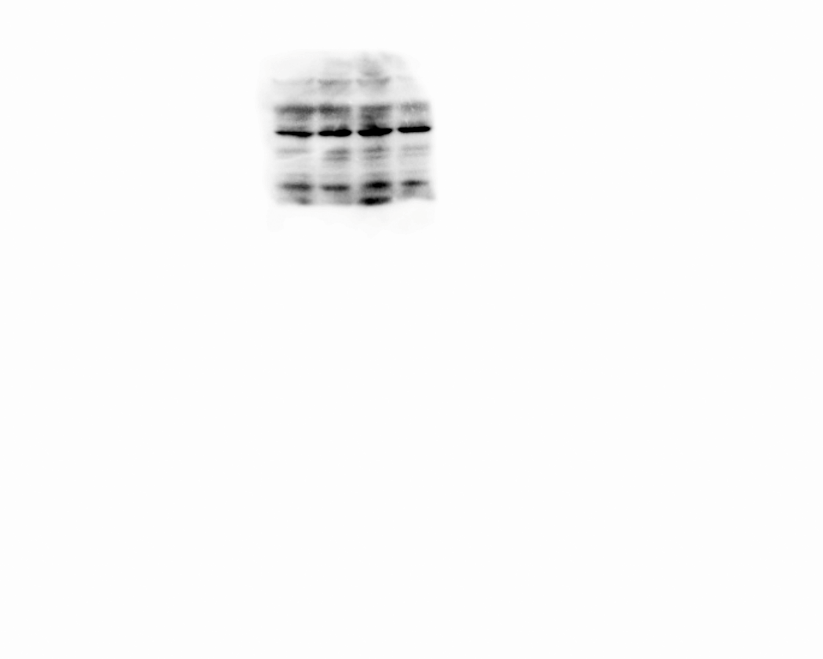


**LPS0Control**

**LPS100Control**

**LPS0siBAFF**

**LPS100siBAFF**

**b**


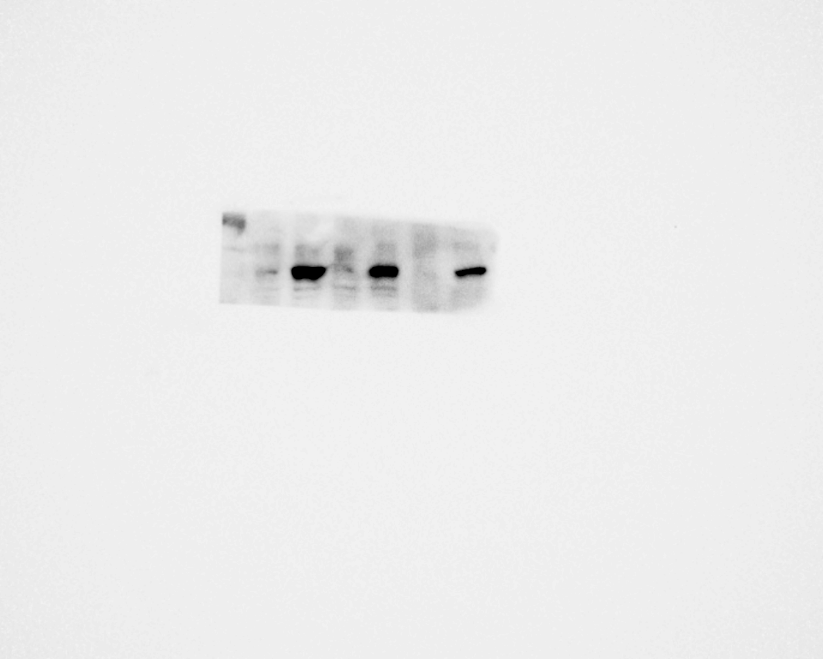


**LPS0Control**

**LPS100Control**

**LPS0siBAFF**

**LPS100siBAFF**

**c**


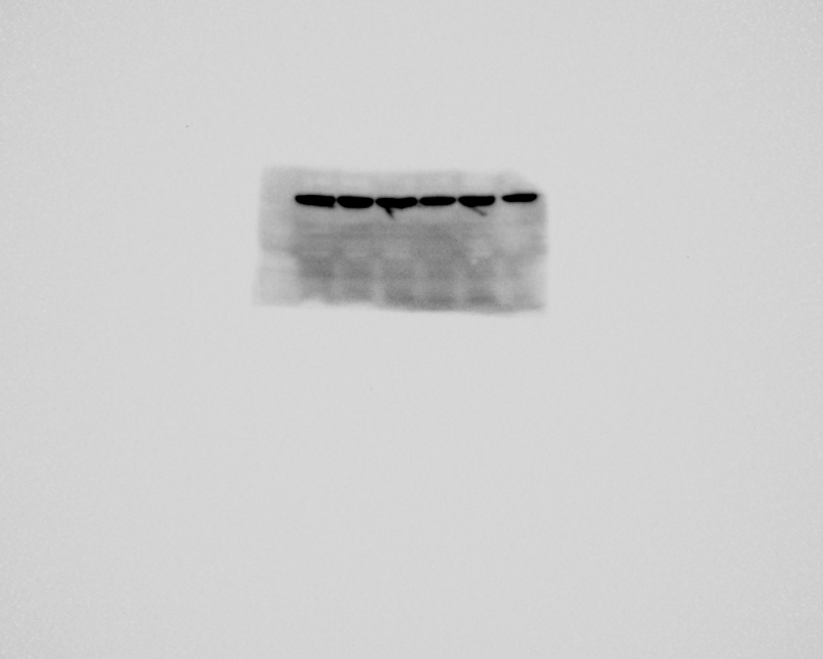


**LPS0Control**

**LPS100Control**

**LPS0siBAFF**

**LPS100siBAFF**

Supplementary Fig. 1 The original, unprocessed images of Western blotting. (a) The original image of Arg1 (M2-related marker) protein. (b) The original image of iNOS (M1-related marker) protein. (c) The original image of β-actin protein.


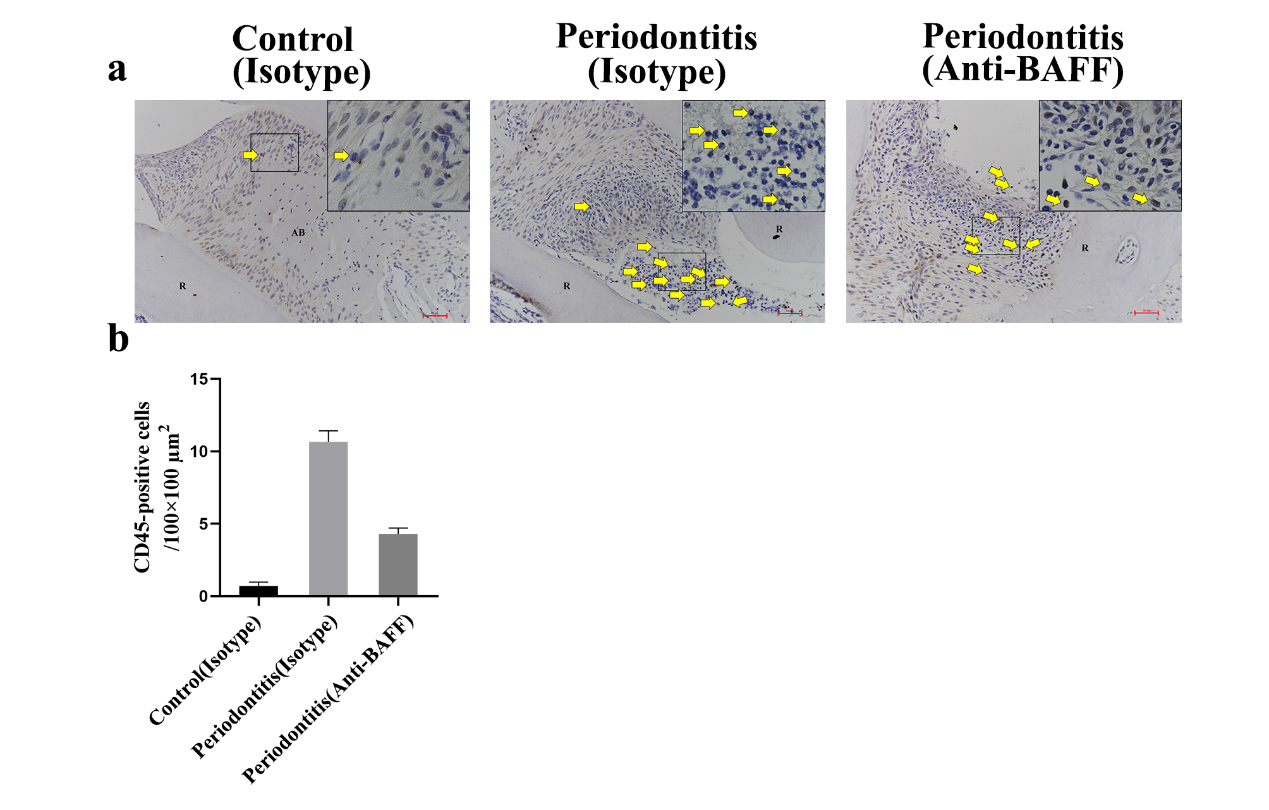


Supplementary Fig. 2 Expression of CD45-positive cells in periodontal tissues of mice. (a) Representative immunohistochemistry micrographs of periodontal tissues of maxillary second molars identifying the leukocytes marker CD45. Yellow arrows indicate areas of high expression of CD45. R, root; AB, alveolar bone. Magnification: 200×. Scale bar, 50 μm. (b) Quantitative analysis of CD45-positive cells /100×100 µm^2^. Data are presented as mean ± standard deviation. *** *P* < 0.001 using one-way ANOVA with Tukey's test. ANOVA, analysis of variance.
